# Cigarette smoke extract profoundly suppresses TNFα-mediated proinflammatory gene expression through upregulation of ATF3 in human coronary artery endothelial cells

**DOI:** 10.1038/srep39945

**Published:** 2017-01-06

**Authors:** Jack E. Teasdale, Georgina G. J. Hazell, Alasdair M. G. Peachey, Graciela B. Sala-Newby, Charles C. T. Hindmarch, Tristan R. McKay, Mark Bond, Andrew C. Newby, Stephen J. White

**Affiliations:** 1 School of Clinical Sciences, University of Bristol, Bristol Royal Infirmary, Bristol, BS2 8HW, UK; 2 Department of Biomedical and Molecular Sciences, Queen’s University, Kingston, ON, Canada, K7L 3N6; 3 Department of Physiology, Faculty of Medicine, University of Malaya, 50603, Kuala Lumpur, Malaysia; 4 School of Healthcare Sciences, Manchester Metropolitan University, Manchester M1 5GD, UK

## Abstract

Endothelial dysfunction caused by the combined action of disturbed flow, inflammatory mediators and oxidants derived from cigarette smoke is known to promote coronary atherosclerosis and increase the likelihood of myocardial infarctions and strokes. Conversely, laminar flow protects against endothelial dysfunction, at least in the initial phases of atherogenesis. We studied the effects of TNFα and cigarette smoke extract on human coronary artery endothelial cells under oscillatory, normal laminar and elevated laminar shear stress for a period of 72 hours. We found, firstly, that laminar flow fails to overcome the inflammatory effects of TNFα under these conditions but that cigarette smoke induces an anti-oxidant response that appears to reduce endothelial inflammation. Elevated laminar flow, TNFα and cigarette smoke extract synergise to induce expression of the transcriptional regulator activating transcription factor 3 (ATF3), which we show by adenovirus driven overexpression, decreases inflammatory gene expression independently of activation of nuclear factor-κB. Our results illustrate the importance of studying endothelial dysfunction *in vitro* over prolonged periods. They also identify ATF3 as an important protective factor against endothelial dysfunction. Modulation of ATF3 expression may represent a novel approach to modulate proinflammatory gene expression and open new therapeutic avenues to treat proinflammatory diseases.

The haemodynamic environment plays an important contributing role in the initiation and progression of atherosclerosis, with disease developing and progressing most rapidly in regions of oscillatory, low and disturbed wall shear stress^1^,^2^,^3^,^4^. Underlying this response is the mechanosensitivity of endothelial cells, whose behaviour is profoundly modulated by the shear environment to which they are exposed. Athero-prone flow (disturbed flow pattern/oscillatory shear stress/low average wall shear stress) provokes endothelial dysfunction, reducing the bioavailability of nitric oxide, while increasing oxidant stress, the magnitude of response to inflammatory cytokines, rates of apoptosis and permeability. The athero-protective phenotype that endothelial cells adopt in normal physiological laminar shear stress (12–16 dynes/cm^2^ in the coronary circulation^5^,^6^,^7^) is predominantly mediated by increased expression of KLF2 and KLF4, and the activation of Nrf2, all of which combine to activate a program of gene expression and epigenetic changes that reduce endothelial dysfunction^8^,^9^. In addition, we have reported that endothelial cells cultured under elevated shear stress (75 dynes/cm^2^) similar to that experienced overlying a stenotic atherosclerotic plaque adopt a unique phenotype^10^, which may therefore react in a different way to mediators of endothelial dysfunction. Under elevated shear stress, endothelial cells amplify many of the features observed at normal physiological flow, further increasing the expression of KLF2 and KLF4^10^, however they have a distinctive transcriptomic and proteomic signature (10 and S. White, unpublished data).

TNFα imposes a significant pro-inflammatory effect on endothelial cells. It induces the expression of adhesion molecules (e.g. VCAM1, ICAM1, E-selectin) and chemokines (e.g. MCP1/CCL2 and fractalkine/CX3CL1)^11^, predominantly through activation of transcription factor NFκB^12^,^13^. TNFα also increases the production of reactive oxygen species (ROS), perpetuating endothelial dysfunction. The responses to TNFα and other inflammatory mediators are ameliorated by shear stress, with KLF2 and Nrf2 inhibition of TNFα activity being part of the anti-inflammatory effects of laminar shear stress^8^,^14^,^15^. Nevertheless, endothelial cells over stenotic atherosclerotic plaques show markers of inflammation^16^, including increased adhesion molecule expression^17^,^18^, implying that the protective effects of flow may be over-ridden in advanced atherosclerosis.

Cigarette use also induces endothelial dysfunction, either indirectly by elevating circulating inflammatory cytokines, including TNFα, or directly by increasing oxidant stress^19^,^20^,^21^,^22^. We have previously demonstrated that fresh aqueous cigarette smoke extract (CSE) induces a stress response in human coronary artery endothelial cells, activating transcription factor Nrf2 and increasing the expression of cytochrome p450 subunits^23^.

In this report we demonstrate the use of our improved model for studying the prolonged effects of oscillatory, physiological and elevated flow. We focus on the interaction of shear stress with the effects of TNFα and CSE as a model of endothelial dysfunction, which contributes to the development of atherosclerosis, our intention being to identify new signalling interactions that might be useful ultimately for future therapy or as biomarkers. Consistent with this aim, we document the shear, TNFα and CSE-dependent upregulation of transcription factor ATF3, identifying a negative-feedback loop that limits proinflammatory gene expression.

## Results

### Effects of flow and cigarette smoke extract (CSE) on TNFα induced gene expression in different, sustained flow environments

**Figure 1:**
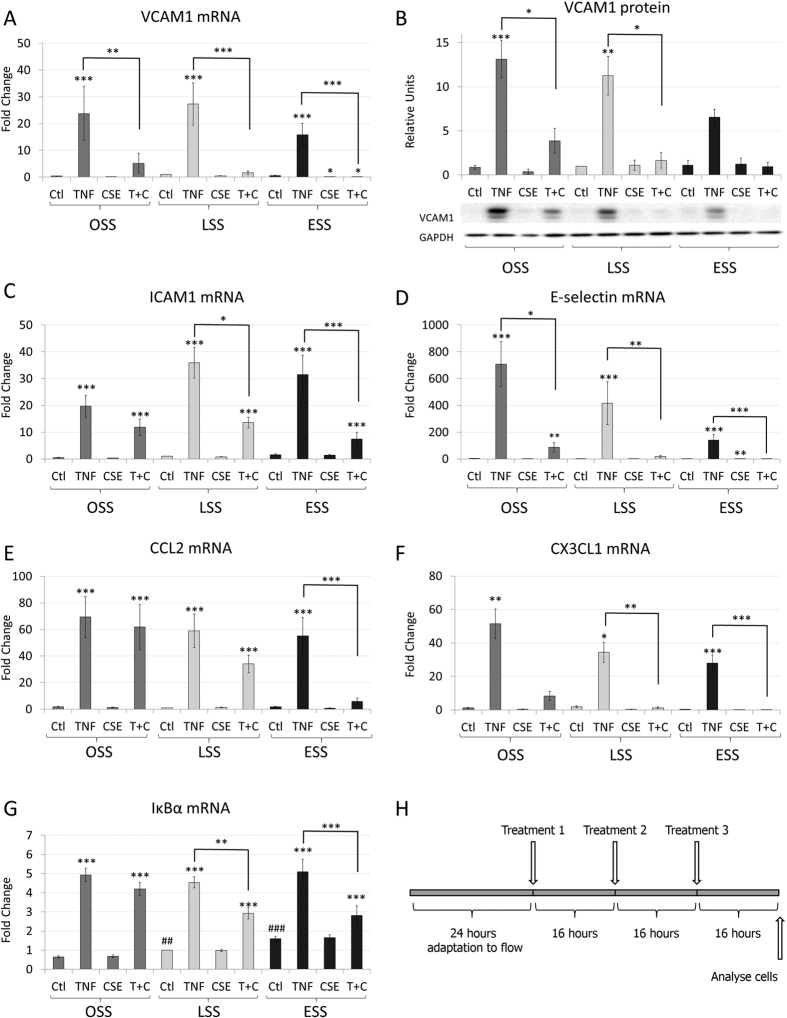
Effects of flow and cigarette smoke extract (CSE) on TNFα induced gene expression. The mRNA expression of (**A**) VCAM1, (C) ICAM1, (**D**) E-selectin, (**E**) CCL2, (**F**) CX3CL1 and (**G**) IκBα, (n = 6 separate batches of HCAECs); or (**B**) protein expression of VCAM-1 (n = 3 separate batches of HCAECs). HCAEC were exposed to 5 ng/ml TNFα, 10% CSE or TNFα and CSE together (T + C) under OSS, LSS or ESS conditions, as illustrated schematically (**H**). Changes are expressed as mean fold change against LSS control ± S.E.M. *p < 0.05; **p < 0.01, ***p < 0.001 significant vs control for that shear stress, or other indicated comparison; ^#^P < 0.05 vs OSS control

We sought to use treatments with TNFα and cigarette smoke extract (CSE), alone or in combination, to mimic pathological effects on human coronary artery endothelial cells (HCAECs). We investigated the effects of sustained exposure under oscillatory (OSS), normal laminar (15 dynes/cm^2^ - LSS) or elevated laminar shear stress (75 dynes/cm^2^ - ESS). OSS models some components of athero-prone flow, LSS models athero-protective flow and ESS models flow overlying stenotic plaques. HCAECs were exposed to shear stress for 24 hours before 3 treatments of 5 ng/ml of TNFα, 10% v/v CSE, or the combination of both treatments were applied to the cells, 16 hours apart (Fig. 1H and supplementary data). The concentrations of mRNAs or proteins were measured 16 hours after the final treatment (48 hours of exposure to stimuli). This protocol allowed the HCAECs time to adapt to the flow environment before treatments were applied and captured the response to sustained exposure to these insults. In agreement with previous findings, TNFα significantly increased the expression of VCAM1 and other intercellular adhesion molecules ICAM1 and E-selectin, as well as cytokines CCL2 and CX3CL1 and the NFκB feed-back regulator, IκBα (Fig. 1A–G). The effect on VCAM-1 was confirmed at the protein level (Fig. 1B). Multiple studies have demonstrated that normal laminar shear stress reduces the magnitude of proinflammatory gene expression induced by TNFα treatment^24^,^25^,^26^,^27^,^28^. We failed to show a significant effect of normal laminar shear stress (LSS) on TNFα-regulated genes, possibly because of the sustained exposure to TNFα. However, we cannot rule out a subtle effect of LSS on TNFα-regulated genes, because the concurrent testing of multiple experimental conditions reduces the power to identify small changes in gene expression. CSE did not increase the expression of any of the pro-inflammatory genes investigated under any flow condition. Surprisingly, however, CSE antagonised the induction of these genes by TNFα under sustained LSS or ESS (Fig. 1A–G). We concluded, firstly, that sustained exposure to TNFα overwhelmed the ability of flow to reduce pro-inflammatory activation. Secondly, CSE appeared to evoke a response that suppressed expression of some pro-inflammatory genes, particularly under normal physiological or elevated laminar shear stress. By contrast, a few of the TNFα responsive genes assayed were not down-regulated by CSE. For example expression of protease inhibitor 3 (PI3) or the p65/RelA subunit of NF-κB were stimulated by TNFα but not suppressed by CSE (Supplementary Figures 4, 5).

### Effects of TNFα and CSE on the expression of shear responsive genes

**Figure 2:**
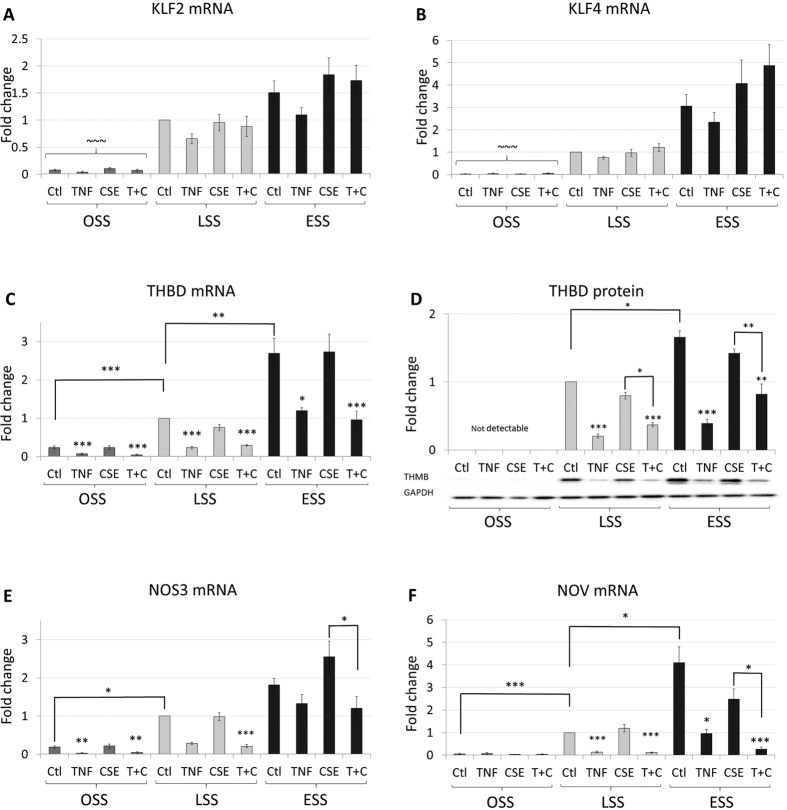
TNFα, but not CSE, suppress the expression of shear-responsive genes. Changes in mRNA expression (n = 6 batches) of (**A**) KLF2, (**B**) KLF4, (C) THBD, (**E**) NOS3, (**F**) NOV or (**D**) THBD protein (n = 3 batches) were measured in HCAECs exposed to 5 ng/ml TNFα, 10% CSE or TNFα and CSE together (T + C) under OSS, LSS or ESS, expressed as mean fold change against control ± S.E.M. *p < 0.05; **p < 0.01, ***p < 0.001 significant vs control for that shear stress, or indicated comparison; ~~~P < 0.001 vs LSS and ESS (all conditions).

Laminar shear stress has been shown to maintain high level expression of multiple athero-protective genes in endothelial cells through induction of transcription factors Kruppel-like factor-2 and 4 (KLF2, KLF4)^8^,^29^. We confirmed these observations in our experiments. For example, KLF2 expression was significantly enhanced in LSS and ESS compared to OSS (13- and 20-fold, respectively Fig. 2A). Similar results were found for KLF4 (33- and 100-fold, respectively, Fig. 2B) and for shear stress responsive genes thrombomodulin (THBD), endothelial nitric oxide synthase (NOS3) and nephroblastoma overexpressed (NOV) (Fig. 2C–F). The effect on the mRNA expression of thromomodulin (THBD) correlated with the protein level (Fig. 2D). The effects of ESS always tended to be greater than LSS but this was only statistically significant for THBD and NOV (Fig. 2C,D,F). Most interestingly, although sustained treatment with TNFα did not affect the upregulation of either KLF2 or KLF4 mRNA, it significantly reduced the expression of THBD mRNA and protein, NOS3 and NOV mRNA. This implies that TNFα can antagonise the transcriptional responses to KLFs, without directly affecting their expression. Sustained treatment with CSE did not affect the upregulation of KLF2 or KLF4 mRNA either, nor did it rescue the TNFα suppression of shear-regulated genes. The anti-inflammatory effects of CSE are therefore unlikely to be mediated through KLF2 or KLF4-dependent mechanisms.

### CSE upregulates the expression of Nrf2 responsive genes at all shear rates

**Figure 3:**
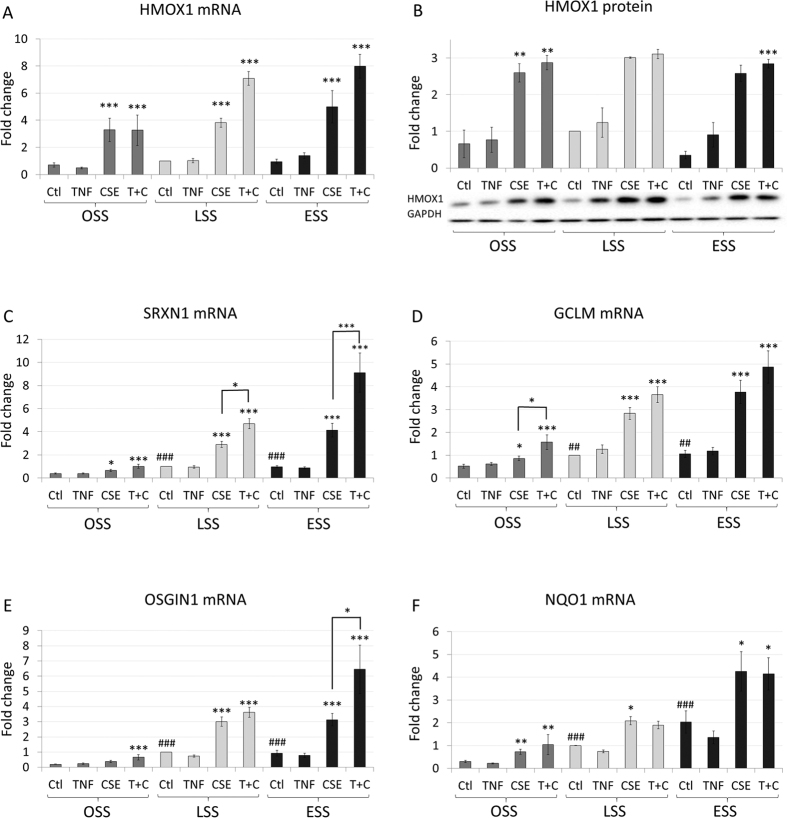
CSE increases the expression of Nrf2 responsive genes. Changes in mRNA expression of (**A**) HMOX1, (**C**) SRXN1, (**D**) GCLM, (**E**) OSGIN1, (**F**) NQO1 (n = 6 batches) or (**B**) HMOX1 protein (n = 3 batches) in HCAECs exposed to 5 ng/ml TNFα, 10% CSE or TNFα and CSE together (T + C) under OSS, LSS or ESS conditions. Changes are expressed as mean fold change against LSS control ± S.E.M. *p < 0.05; **p < 0.01, ***p < 0.001 significant vs control for that shear stress, or indicated comparison; ^#^P < 0.05 vs OSS control.

The transcription factor, Nrf2, is central to the antioxidant response in endothelial cells^15^,^30^,^31^,^32^, having been shown to increase the expression of a group of genes that includes heme oxygenase 1 (HMOX1)^23^. Administration of cigarette smoke extract (CSE) significantly increased HMOX1 expression to a similar extent at mRNA and protein levels in all the shear environments we tested (Fig. 3A,B). CSE also increased the expression of the other antioxidant response genes (which are validated Nrf2 targets^23^,^33^) in HCAECs and in most cases the effects were amplified at the higher shear rates. By contrast, TNFα alone had no effect on any Nrf2 target gene studied at any shear rate (Fig. 3A–E). However, the addition of TNFα to CSE significantly enhanced the expression of sulfiredoxin 1 (SRXN1) and oxidative stress induced growth inhibitor 1 (OSGIN1) at ESS. The anti-inflammatory effects of CSE could therefore be mediated by an Nrf2-dependent process.

### Shear stress, TNFα and CSE modulate ATF3 expression

**Figure 4:**
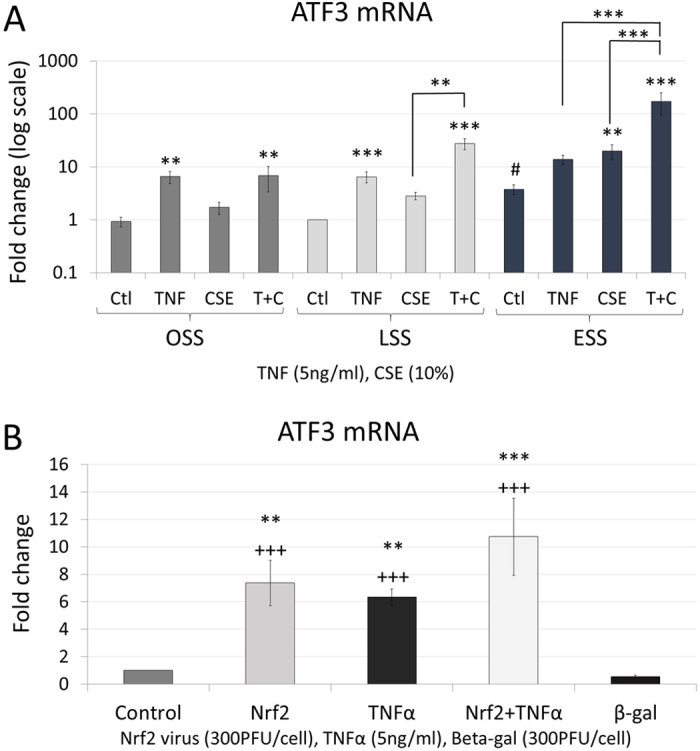
Shear stress, TNFα and CSE all increase ATF3 expression. Changes in (**A**) ATF3 mRNA expression in HCAECs exposed to 5 ng/ml TNFα, 10% CSE or TNFα and CSE together (T + C) under OSS, LSS or ESS, expressed as mean fold change against control ± S.E.M. *p < 0.05; **p < 0.01, ***p < 0.001 significant vs control for that shear stress, or indicated comparison; ^#^P < 0.05 vs OSS control; n = 6 batches. (**B**) Regulation of ATF3 mRNA expression in HCAECs following transduction with adenoviral vector overexpressing Nrf2, treatment with 5 ng/ml TNFα, or the combination in static culture. **P < 0.01 vs Untreated Control; ^+++^P < 0.001 vs β-gal adenoviral vector control.

Transcription factor, ATF3, was previously shown to inhibit Toll-like receptor 4 (TLR4)-stimulated inflammatory response by repression of NFκB responsive genes in macrophages^34^, potentially by localising inhibitory co-factors at the promoter. Therefore we investigated whether ATF3 expression was modulated in our system and could act as a suppressor of pro-inflammatory gene induction. LSS did not significantly increase ATF3 mRNA expression but ESS increased it 3.7-fold compared to LSS (Fig. 4A, note the logarithmic scale). CSE did not increase ATF3 expression at OSS or LSS but increased it at ESS (an additional 5-fold above ESS control Fig. 4A). TNFα tended to increase ATF3 expression at all shear stresses and this was significant at OSS and LSS (6.5-fold increase). CSE and TNFα produced synergistic effects at LSS and ESS (approximately 10-fold increases above CSE alone Fig. 4A). Hence, CSE, TNFα and ESS were all necessary to maximise the expression ATF3 in HCAECs. Importantly, the highest level of ATF3 expression (175-fold above LSS alone and 185-fold increase over OSS) was observed under the same conditions where proinflammatory gene expression showed the greatest inhibition (Fig. 1).

To investigate the regulation of ATF3, we used adenoviral overexpression of Nrf2 in static cultures of HCAECs. Adenoviral Nrf2 overexpression increased ATF3 expression. Consistent with the experiments under flow (Fig. 4A), TNFα also stimulated ATF3 expression in static cultures (Fig. 4B). The effects of Nrf2 overexpression and TNFα stimulation were less than additive.

### ATF3 reduces proinflammatory gene expression without reducing NFκB activation, nuclear entry, DNA binding or activation

**Figure 5:**
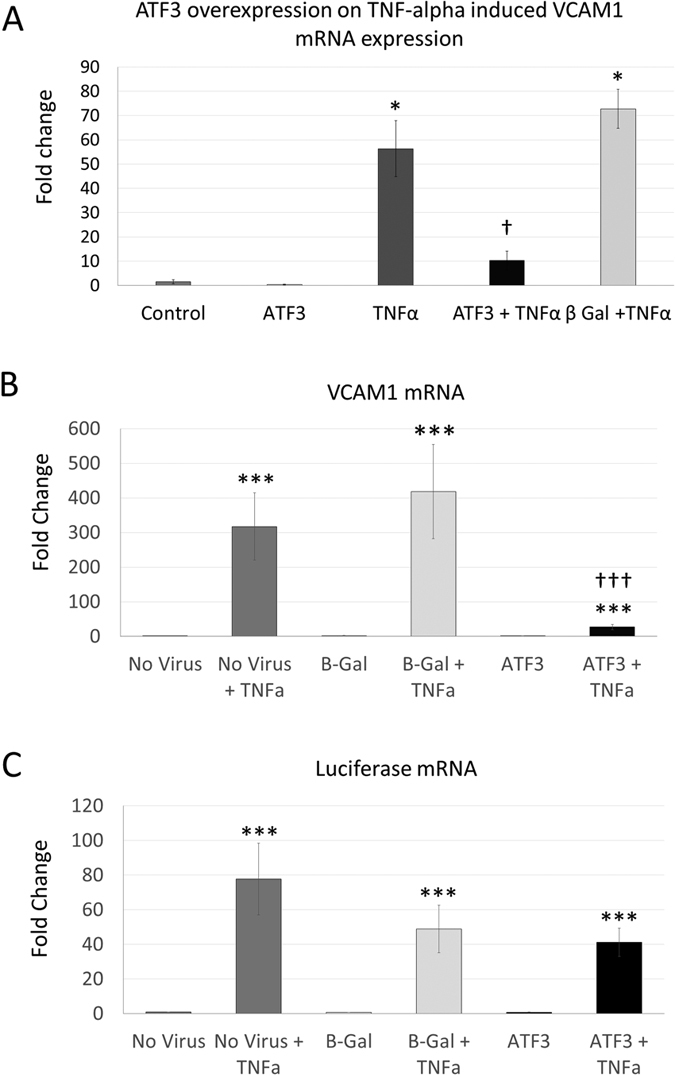
ATF3 overexpression reduces VCAM1 upregulation. (**A**) Adenoviral-mediated ATF3 overexpression reduced TNFα-stimulated VCAM1 upregulation in HCAEC. (**B**) In NFκB reporter endothelial cell line NF-EA.hy926, ATF3 overexpression reduced TNFα-stimulated VCAM1 mRNA upregulation; however did not inhibit NFκB reporter activity (**C**), measured by changes in luciferase mRNA expression. *P < 0.05 vs untreated control; ^†††^P < 0.001 vs no virus + TNFα or β-gal viral control + TNFα; n = 3.

We hypothesised that ATF3 may play a role in the down-regulation of TNFα stimulated pro-inflammatory genes. Consistent with this, adenoviral-mediated ATF3 overexpression significantly reduced the level of VCAM1 expression when HCAECs were stimulated with TNFα in static culture (Fig. 5A). ATF3 overexpression also altered the morphology of HCAECs, inducing an elongation, with no sign of induction of cell stress or apoptosis (Supplementary Figure 6). To investigate whether ATF3 interfered with NFκB activation or activity, we created a lentiviral-based NFκB luciferase reporter^35^ expression construct in endothelial cell line EA.hy926 (NF-EA.hy926). In NF-EA.hy926 cells, ATF3 overexpression again inhibited the TNFα upregulation of VCAM1 (Fig. 5B), but there was no effect of ATF3 overexpression on the TNFα-dependent upregulation of luciferase (Fig. 5C), indicating that ATF3 overexpression did not prevent NFκB activation, nuclear entry, DNA binding and ability to activate transcription of the reporter.

## Discussion

Our model allowed HCAEC to adapt to their shear environment for 24 hours before treatment. Under these conditions, repeated exposures to TNFα over a 48 hour period upregulated the expression of a panel of NFκB responsive genes. Importantly, the anti-inflammatory effects of laminar shear that have been reported at shorter time points in other models do not apparently persist after longer exposures to TNFα in our model. Instead, this study identified an unexpected inhibitory effect of repeated exposure to CSE on expression of a subset of pro-inflammatory genes. This effect correlated with the upregulation of Nrf2 rather than KLF2/4. Furthermore, TNFα, laminar shear and CSE synergised to induce the transcriptional inhibitor ATF3, which we showed to be an Nrf2 responsive gene. We found that ATF3 expression in HCAECs has a large dynamic range, with its expression increasing 185-fold between the highest and lowest conditions. Overexpression of ATF3 was shown to inhibit pro-inflammatory gene expression but independently of a direct effect on NFκB activity. ATF3 upregulation therefore constitutes an NFκB-independent protective mechanism that limits the expression of proinflammatory genes.

ATF3 is upregulated in a multitude of stressed tissues^36^, hypoxic conditions^37^ and some, but not all cancers^38^,^39^,^40^, where its actions promote metastasis^41^. As a homodimer, ATF3 binds to the cyclic AMP response element and represses transcription^42^, potentially by localising inhibitory co-factors at the promoter. Gilchrist *et. al.* demonstrated that, in macrophages, ATF3 expression was induced by LPS treatment and inhibited TLR4-stimulated inflammatory responses as part of a negative-feedback loop^34^. Interestingly, they showed the predominant inhibitory action was seen where ATF3 binding sites closely apposed NFκB binding sites, which was supported by chromatin immunoprecipitation experiments. We therefore, think it is most likely that ATF3 upregulation and inhibition of NFκB-mediated transcription, is the mechanism responsible for the anti-inflammatory effect elicited by CSE in HCAEC. Furthermore, macrophages from ATF3^−/−^ mice responded more vigorously to LPS treatment and suffered greater mortality to LPS challenge, suggesting a general role for ATF3 in dampening inflammation. Consistent with these results, we observed that adenoviral-mediated overexpression of ATF3 inhibited TNFα-dependent VCAM1 expression. This may be context specific as another group demonstrated that ATF3, in concert with TGFβ signalling^43^, increased pro-inflammatory gene expression. The action of NFκB is very context specific and regulates both prosurvival and proinflammatory gene expression^44^, further work is needed to identify which NFκB responsive genes are subject to ATF3-mediated repression. KLF4 was also shown to repress VCAM1 expression^45^, but because CSE did not alter KLF4 mRNA levels or downstream gene expression, it is unlikely that KLF4 contributes to the anti-inflammatory effects of CSE.

In contrast to previous reports, we did not observe a reduction in KLF2 or KLF4 expression with TNFα treatment. NFκB triggered the recruitment of HDAC4 and 5 to the promoter of KLF2 reducing its expression in static culture in HUVEC^46^. This suggests that prolonged shear stress may reduce the recruitment of HDAC4 and 5 to the promoter of KLF2, or that there is a difference in the response of HUVEC and HCAEC to TNFα treatment. Additionally, while TNFα induced an increase in ROS production (sup Fig. 3A), this ROS did not activate Nrf2 responsive genes (Fig. 3). This suggests a specific localisation or type of ROS is necessary for Nrf2 activation. CSE is known to contain very high levels of free radicals^47^,^48^, indicating Nrf2 activation is much more sensitive to this rather than endogenously generated ROS. There is also the possibility of cell-specific effects in this regard, as TNFα was shown to activate Nrf2 in macrophages^49^.

Our data hints at the potential of using local upregulation of ATF3 as a new avenue for treating inflammatory diseases. While the detrimental effects of smoking are well documented (tobacco contributes to the deaths of up to half of its users – World Health Organisation statistics), there are a few pathologies where it has been shown that smoking reduces the severity of the symptoms. For example, ulcerative colitis^50^, recurrent aphthous stomatitis^51^, Behçet’s disease^52^ (which is noted to flare up on cessation of smoking) and Parkinson’s disease^53^ (where smoking has a protective effect on the development of symptoms). While the detrimental effects of smoking far outweigh any beneficial effects, our work suggests that part of the protective effect in these select group pathologies might be mediated through ATF3 upregulation by cigarette smoke; however further investigation would be needed to support this hypothesis. Directly augmenting ATF3 expression, using localised gene transfer or application of Nrf2 agonists^54^,^55^,^56^, could offer therapeutic potential by bypassing the damaging effects of free radicals generated in tobacco combustion.

Our *in vitro* model has the advantage that endothelial cells have time to adapt to their shear environment before treatments are applied. This prevents any treatment from interfering with the adaptation process, which might be a confounding factor where the treatments are applied at the commencement of flow. The extended nature of these flow experiments does however preclude pre-treatment with agents that limit endothelial proliferation, as we aim to start with a completely confluent monolayer of cells. CSE was generated by bubbling filtered mainstream smoke through tissue culture media^23^. This captured the water-soluble components of cigarette smoke, which was filtered to remove particulate matter and immediately applied to cells, to prevent the quenching of dissolved free radicals. The water-soluble components of cigarette smoke have greater relevance for the study of the systemic effects of cigarette smoke, although it is not possible to assess if CSE generated in this way captures the same components of cigarette smoke that are capable of passing through the lungs into the blood stream. In addition, the CSE is administered to cells as a bolus every 16 hours, because of the experimental complexity of administering smaller doses at shorter time intervals. This very approximately equates to an average person smoking 25 cigarettes^23^.

In conclusion, this study has identified a novel anti-inflammatory pathway in HCAECs that is synergistically activated by TNFα, laminar shear and aqueous CSE and is capable of significantly downregulating adhesion molecule and inflammatory cytokine expression. Furthermore, this effect can be recapitulated by the upregulation of transcription factor ATF3.

## Methods

### Cell culture

Human coronary artery endothelial cells (HCAECs, PromoCell, passage 3 to 6) were cultured in endothelial cell growth medium kit (MV2, PromoCell) with the addition of 100 U/ml penicillin/streptomycin. For flow experiments, HCAECs were seeded at 2. 5 × 10^5^ cells/slide onto gelatin coated glass slides within sterile silicon gaskets that limited cell attachment to the central portion of the slide (~9 cm^2^). Cells were grown within the gaskets on the slides for a minimum of 72 hours prior to flow to ensure a completely confluent monolayer and allow maturation of cell-cell and cell-matrix interactions. The slide was assembled aseptically into a previously described parallel plate flow apparatus^10^ for exposure to oscillatory (OSS, 0 ± 5 dynes), laminar shear stress −15 dynes/cm^2^ (LSS), or elevated laminar shear stress −75 dynes/cm^2^ (ESS) for 72 hours. Cells were exposed to shear stress for 24 hours prior to any treatment to allow them to adapt to their shear environment and adopt the relevant phenotype. Cigarette smoke extract (CSE) was prepared, filter sterilized using 0.20 μm filter and used immediately upon creation as described^23^. Briefly, CSE was created using ~385 ml of mainstream smoke (7–8 average puffs) from a single Marlboro Gold cigarette (7 mg tar, 0.6 mg nicotine) drawn through 10 ml of media MV2 at a rate of 70 ml/min. HCAECs were treated with 5 ng/ml tumour necrosis factor-alpha (TNFα, Milteny Biotech) and/or 10% CSE for 48 hours (total of 3 treatments, one every 16 hours, with cells analysed 16 hours after the last treatment, see Fig. 1H). For all flow experiments, MV2 media was made up omitting hydrocortisone and additional ascorbic, acid and supplemented with 3.35% dextran (31390-Sigma) to raise the viscosity to 1.5 cP, equivalent to plasma.

### Viral transduction

Wild type Nrf2 adenovirus (AdNrf2^15^) was generous gifts from Professor Paul Evans (Sheffield University, UK). ATF3 overexpressing adenovirus (AdATF3) was created by PCR amplification of cDNA with SW706F/707 R (See the online supplement), and cloned into *BglII*/*NheI* sites of CpG-free-mcs (Invivogen). The whole expression cassette was then shuttled into pDC511 (Microbix Biosystems, Canada) by *EcoRI* digest. The β-galactosidase (β-gal) control adenovirus (Adβ-gal) was made by cloning the *EcoRI* digest fragment containing the same expression cassette and β-galactosidase gene from CpG-LacZ (Invivogen) into pDC511. Both were then produced using the Microbix Biosystems kit according to their protocols. Optimal transduction of HCAEC was achieved using 300 PFU/cell overnight. NFκB reporter cell line NF-EA.hy926 was created by transducing the endothelial cell line EA.hy926 with a lentiviral vector containing 8 repeats of a NFκB binding site (GGGACTTTCC) minimal promoter and FLAG-Luc-2A-eGFP insert as described^35^.

### Western Blotting

Western blots were performed on SDS protein lysates quantified by picogreen assay, as described^10^. Lysates that contained 10,000 cells were resolved on SDS polyacrylamide gels, before transferring onto PVDF membranes and probing with primary antibodies overnight at 4 °C. Antibodies used: VCAM1 (AF643, R&D), THMB (Ab109189, Abcam), HMOX1 (Ab52947, Abcam), NFκB (#8242, Cell Signalling), NFκB pSer536 (#3033, Cell Signalling).

### Quantitative PCR

Quantitative PCR was performed using the Roche SYBR green PCR mastermix on cDNA prepared using the QuantiTect Reverse Transcription Kit (Qiagen). Primers are listed in the supplementary methods.

### Statistics

Statistical analyses were performed using a one way ANOVA with Tukey-Kramer post hoc tests, or two-way ANOVA for experiments combining shear stress and exposure to TNFα or CSE, with Bonferroni post-test. *p < 0.05; **p < 0.01, ***p < 0.001.

## Additional Information

**How to cite this article**: Teasdale, J. E. *et al*. Cigarette smoke extract profoundly suppresses TNFa-mediated proinflammatory gene expression through upregulation of ATF3 in human coronary artery endothelial cells. *Sci. Rep.* **7**, 39945; doi: 10.1038/srep39945 (2017).

**Publisher's note:** Springer Nature remains neutral with regard to jurisdictional claims in published maps and institutional affiliations.

## Supplementary Material

Supplementary Information
