# Supplementary material for: Cigarette smoke extract profoundly suppresses TNFα-mediated proinflammatory gene expression through upregulation of ATF3 in human coronary artery endothelial cells
Source: Sci Rep. 2017 Jan 6;7:39945. doi: 10.1038/srep39945 (PMC5216376; doi:10.1038/srep39945)
Supplement: Supplementary Information [file srep39945-s1.pdf]

Supplementary data

Cigarette smoke extract profoundly suppresses TNFα-mediated proinflammatory gene expression through upregulation of ATF3 in human coronary artery endothelial cells

Jack E. Teasdale[1], Georgina G. J. Hazell[1], Alasdair M. G. Peachey[1], Graciela B. Sala-Newby[1], Charles C. T. Hindmarch[2], Tristan R. McKay[3], Mark Bond[1], Andrew C. Newby[1] and Stephen J. White[3*].

TNFα strongly upregulates VCAM1 expression in static cultures of ECs[1] and we previously confirmed this in HCAECs[2], defining 5 ng/ml as a maximally effective concentration and 16 hours as an optimal time point (supplementary figures 1, 2). Additionally, TNFα caused a sustained increase in intracellular ROS production, measured in HCAECs 24 hours post treatment (supplementary figures 3A, 3B). While CSE did not increase intracellular ROS production, it significantly enhanced the effect of TNFα alone (Supplementary figure 3C). Neither treatment caused any increase in apoptosis measured by PARP cleavage, nor was there detectable cytotoxicity (ref[2] and associated supplementary data). Based on these data, 5 ng/ml of TNFα and 10% v/v CSE were used in subsequent experiments

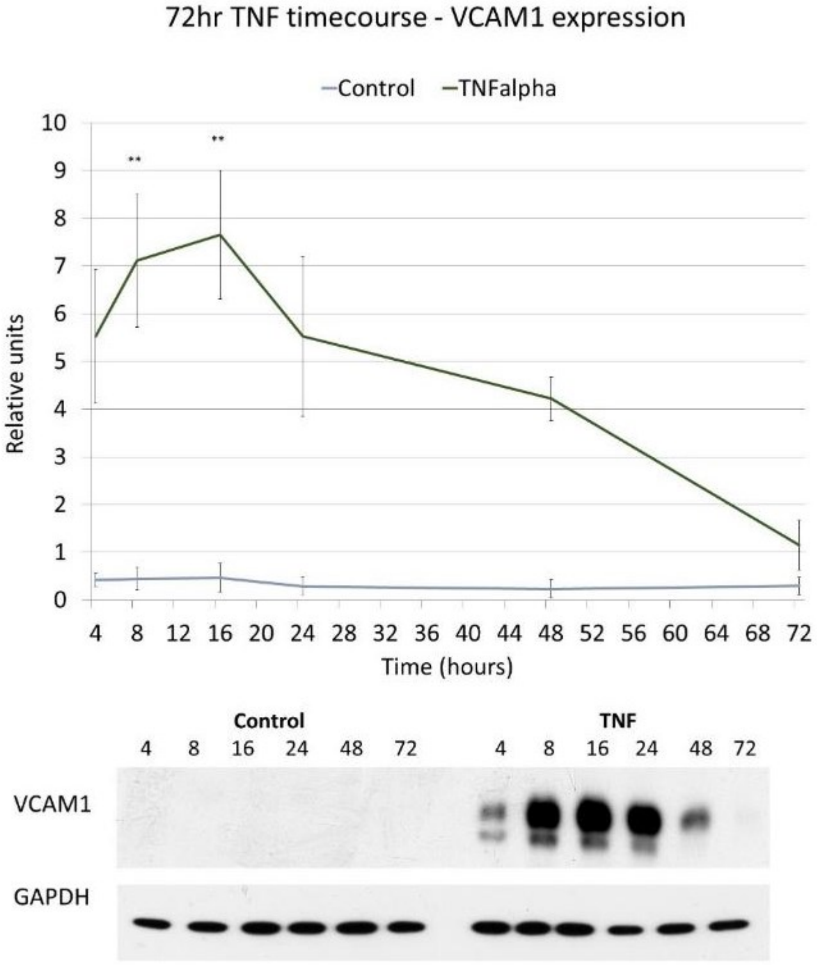

Supplementary figure 1. A time course of VCAM1 protein expression in static HCAECs exposed to TNFα over 72 hours. VCAM1 protein expression was quantified using western blot analysis, expressed as mean fold change against control ± S.E. n=3. ** P<0.01 vs control.

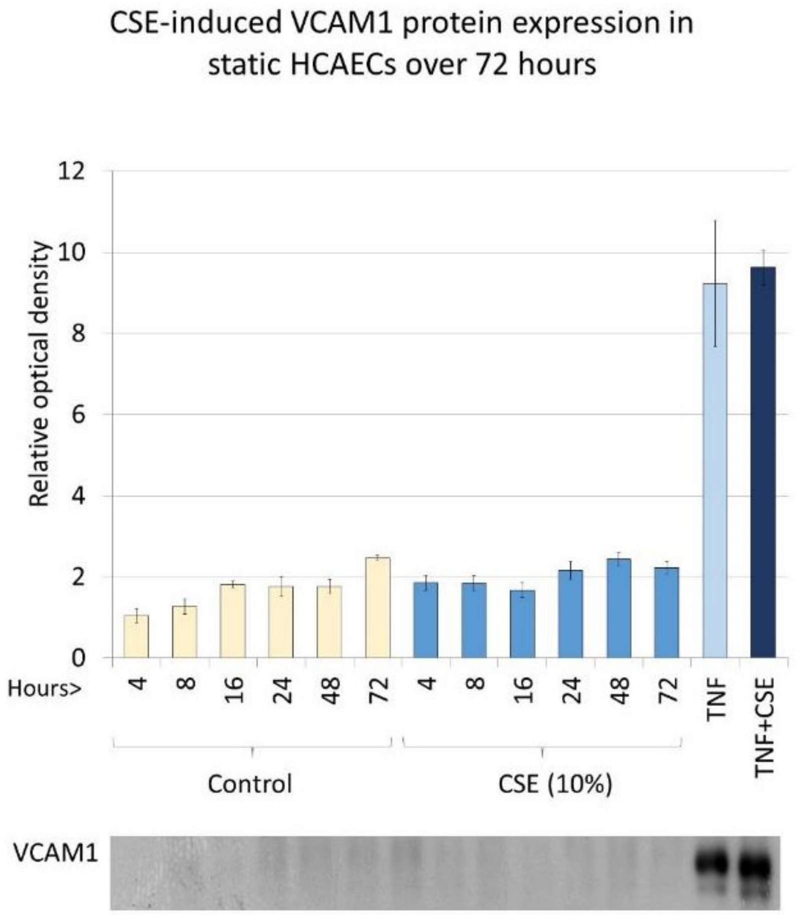

Supplementary figure 2. VCAM1 protein expression in static HCAECs exposed to CSE over 72 hours, with TNFα or TNFα + CSE positive controls. VCAM1 protein expression was quantified using western blot analysis, expressed as mean fold change against control ± S.E. n=3.

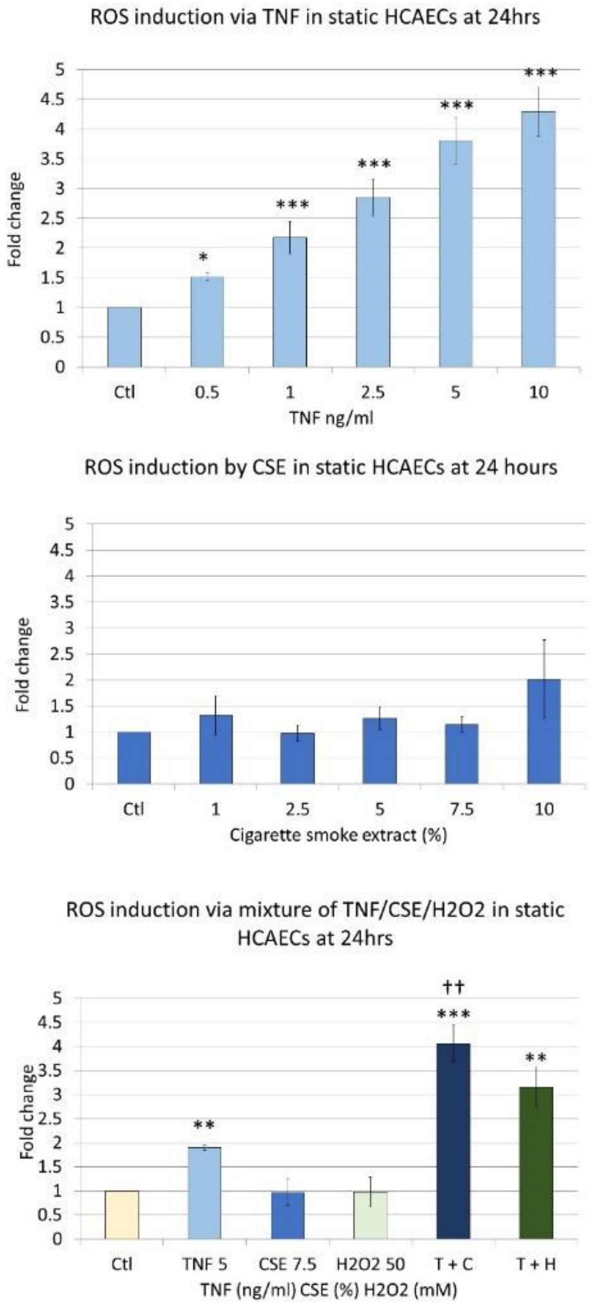

Supplementary figure 3. Induction of reactive oxygen species in static HCAECs exposed to TNFα and CSE. A, 24hr exposure to TNFα in static HCAECs, ROS expression was quantified using DCF-DA fluorescence, expressed as mean fold change against control ± S.E. n=4. * $P<0.05$ compared to control; *** $P<0.001$ compared to control. B, 24hr exposure to CSE in static HCAECs. ROS expression was quantified using DCF-DA fluorescence, expressed as mean fold change against control ± S.E. n=4. C. Induction of reactive oxygen species in static HCAECs exposed to TNFα, CSE and H_2O_2 and their combination. ROS expression was quantified using DCF-DA fluorescence, expressed as mean fold change against control ± S.E. n=4.* $P<0.05$ compared to supplemented control; †† $P<0.01$ vs TNFα alone.

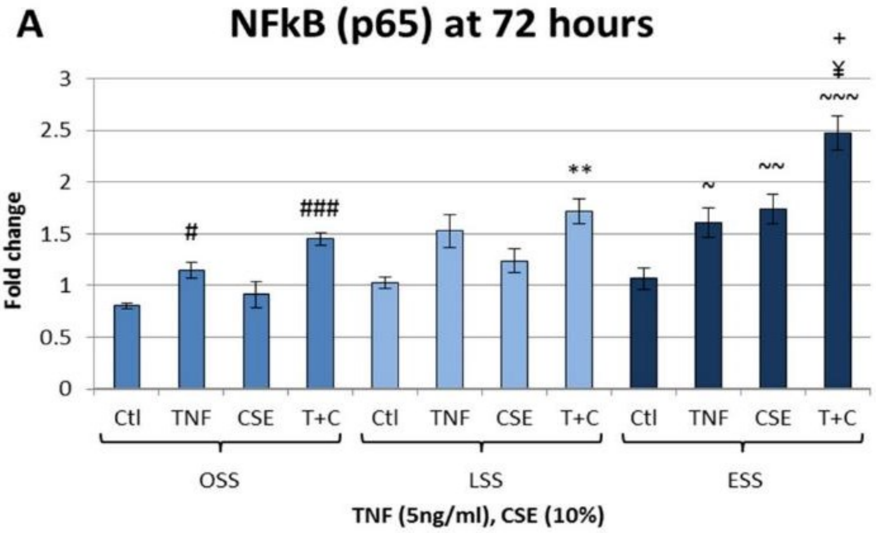

Supplementary figure 4. NFκB (P65) mRNA expression in HCAECs exposed to TNFα, CSE or TNFα and CSE together (T+C) under OSS, LSS or ESS conditions for 72 hours. NFκB p65 mRNA expression was quantified using western blotting analysis, expressed as mean fold change against control ± S.E. n=3. ### P<0.001 vs OSS control; ** P<0.01 vs LSS control; ~~~ P<0.001 vs ESS control; ¥ P<0.05 vs ESST; + P<0.05 vs ESSC.

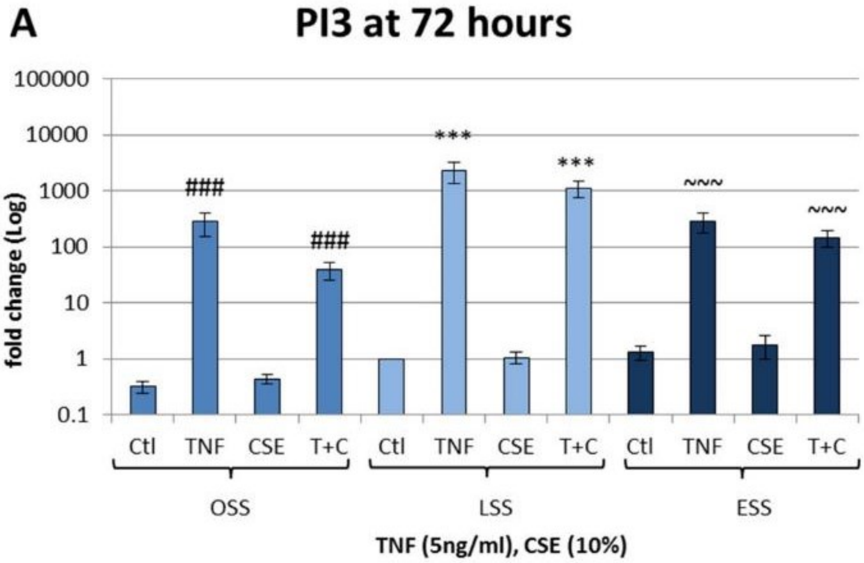

Supplementary figure 5. PI3 mRNA expression in HCAECs exposed to TNFα, CSE or TNFα and CSE together (T+C) under OSS, LSS or ESS conditions for 72 hours. PI3 mRNA expression was quantified using real-time PCR, expressed as mean fold change against control ± S.E. n=6. ### P<0.001 vs OSS control; *** P<0.001 vs LSS control; ~~~ P<0.001 vs ESS control. n=6

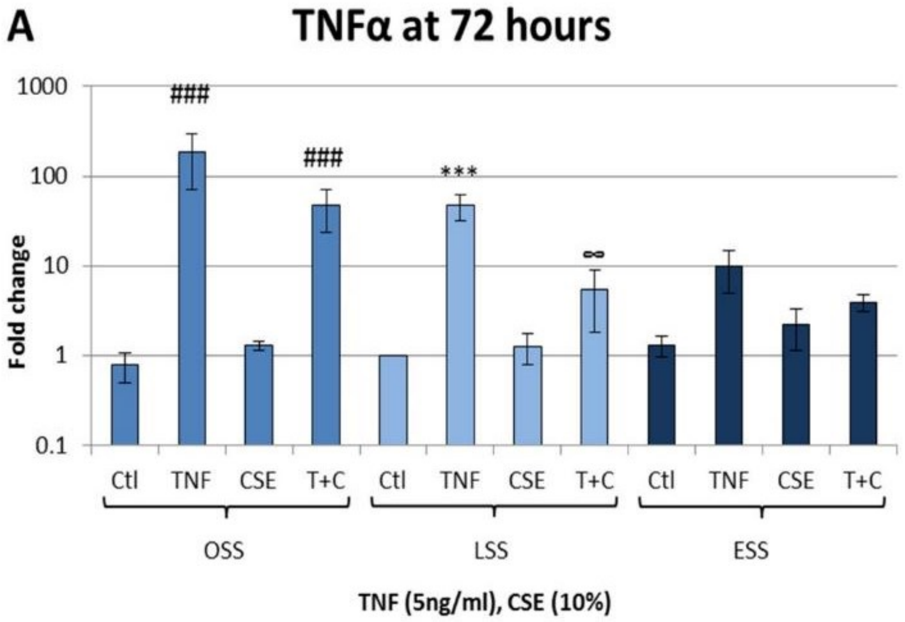

Supplementary figure 6. TNFα mRNA expression in HCAECs exposed to TNFα, CSE or TNFα and CSE together (T+C) under OSS, LSS or ESS conditions for 72 hours. TNFα mRNA expression was quantified using real-time PCR, expressed as mean fold change against control ± S.E. n=6. ### P<0.001 vs OSS control; *** P<0.001 vs LSS control; ∞ P<0.05 vs LSST. n=6

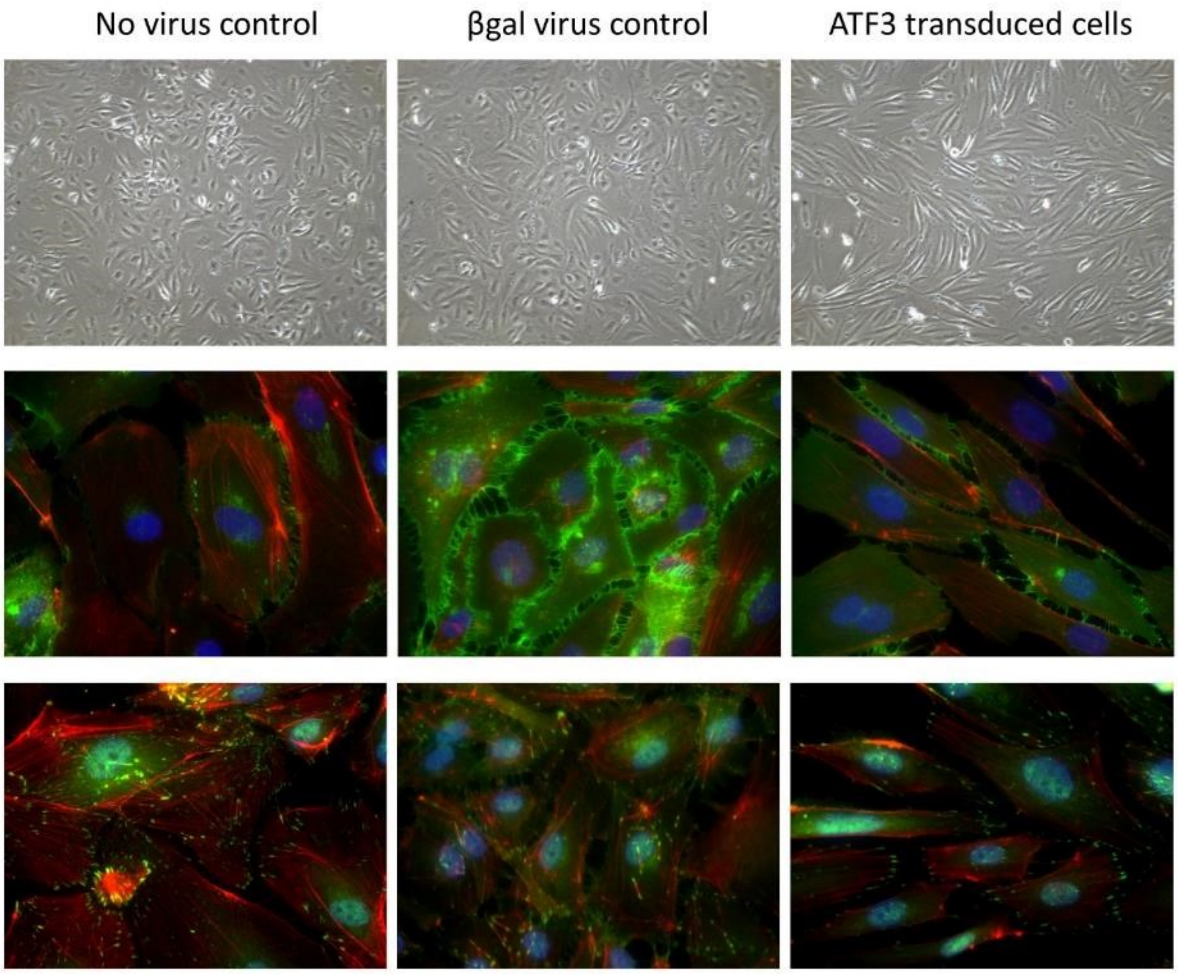

Supplementary figure 7. ATF3 overexpression consistently induced a morphological change in endothelial cells. A, B, C – phase contrast images of HCAEC, A) no virus control; B) βgal virus control (300pfu/cell) C) AdATF3 (300pfu/cell). D, E, F – VE-cadherin (green) and Phalloidin (Red). G, H, I – Vinculin (green) and Phalloidin (Red).

Supplementary methods

Primer sequences

Code	Gene	Sequence	Anneal (°C)	Extension (Sec)
SW381	ATF3	CCCTCCTGGGTCACTGGTGT	62	15
SW382	ATF3	CTTCAGGGGCTACCTCGGCT	62	15
SW706F	ATF3-cloning	GCGAGATCTGGAGCACCATGATGCTTCAACACCC	65	60
SW707R	ATF3-cloning	CACGCTAGCTTAGCTCTGCAATGTTCCTTC	65	60
SW704F	CCL2	ATTCCCCAAGGGCTCGCTCAG	62	15
SW705R	CCL2	ACTTCTGCTTGGGGTCAGCACA	62	15
SW718	CX3CL1	CTGTCGTGGCTGCTCCGCTT	62	15
SW719	CX3CL1	TCGGGTCGGCACAGAACAGC	62	15
SW204	eNOS	GCCGGAACAGCACAAGAGT	60	30
SW205	eNOS	GAGGATGCCAAGGCCGC	60	30
SW767	E-SELECTIN	CTGGGCTCCAGGTGAACCCAAC	62	20
SW768	E-SELECTIN	CCGTGGCCACTGCAGGATGTA	62	20
SW180	GAPDH	CGGATTTGGTCGTATTGGGCG	60	30
SW181	GAPDH	GCCTTCTCCATGGTGGTGAAGAC	60	30
SW754	GCLM	GTCCTTGGAGTTGCACAGCTGGA	62	20
SW755	GCLM	GGCATCACACAGCAGGAGGCA	62	20
SW226F	HMOX1	TCAGGCAGAGGGTGATAGAAGAGG	60	30
SW227R	HMOX1	GCCACCAGAAAGCTGAGTGTAAGG	60	30
SW252	ICAM1	CTAAAGGATGGCACTTTCCCACTG	62	20
SW253	ICAM1	CCTTTTTGGGCCTGTTGTAGTCTG	62	20
SW740	IκBα	CGCCCAAGCACCCGGATACA	62	20
SW741	IκBα	AACGTCAGACGCTGGCCTCC	62	20
SW218	KLF2	GTGAGAAGCCCTACCACTGCAACT	60	30
SW219	KLF2	CCGGTTCTCTGGGTCCAATAAATA	60	30
SW369	KLF4	TGGACCCCCTCTCAGCAATG	60	30
SW370	KLF4	CTCTTGGTAATGGAGCGGCG	60	30
SW771	NFκB (RelA)	GAAAGGACTGCCGGGATGGCT	62	20
SW772	NFκB (RelA)	GTAGTCCCCACGCTGCTCTTCT	62	20
SW716	NOV	TGGTGCGGCCCTGTGAACAA	62	15
SW717	NOV	AGCGGCCATCACTGCAGACC	62	15
SW496F	NQO1	CTAGTTCCGGCCAGGGTCGC	62	20
SW497R	NQO1	TCCGACTCCACCACCTCCCA	62	20
SW748	OSGIN1	GGGAGCCTGGCACTCCATCG	62	15
SW749	OSGIN1	CCCGGCTGTTGCGAAGACCT	62	15
SW671	PI3	CCTCATCGCTGGGACGCTGG	62	15
SW672	PI3	GGGCAGGAGCCAGGCTTAGT	62	15
SW756	SRXN1	GCCAAGGTGCAGAGCCTCGT	62	15
SW757	SRXN1	GCGGGGATGGTCTCTCGCTG	62	15

SW373	THMB	CAACACACAGGGTGGCTTCG	60	30
SW374	THMB	GGCTGGACAGGCAGTCTGGT	60	30
SW541	TNFα	TCGAACCCCGAGTGACAAGCC	62	20
SW542	TNFα	CTGGTAGGAGACGGCGATGCG	62	20
SW232	VCAM1	GAACCCAAACAAAGGCAGAGTACG	60	30
SW233	VCAM1	TGCTTCTTCCAGCCTGGTTAATTC	60	30

1 Wellicome, S. M. *et al.* A monoclonal antibody that detects a novel antigen on endothelial cells that is induced by tumor necrosis factor, IL-1, or lipopolysaccharide. *J. Immunol* **144**, 2558-2565 (1990).

2 Teasdale, J. E., Newby, A. C., Timpson, N. J., Munafò, M. R. & White, S. J. Cigarette smoke but not electronic cigarette aerosol activates a stress response in human coronary artery endothelial cells in culture. *Drug Alcohol Depend.* **163**, 256-260, doi:http://dx.doi.org/10.1016/j.drugalcdep.2016.04.020 (2016).
